# *elk1*/miR-462-731 Feedback Loop Regulates Macrophages Polarization and Phagocytosis in Grass Carp (*Ctenopharyngodon idella*)

**DOI:** 10.3389/fimmu.2022.946857

**Published:** 2022-07-14

**Authors:** Yan He, Yuting Liu, Yuyue Yang, Yang Liu, Xuewen Jia, Yubang Shen, Xiaoyan Xu, Jiale Li

**Affiliations:** ^1^ Key Laboratory of Freshwater Aquatic Genetic Resources Ministry of Agriculture and Rural Affairs, Shanghai Ocean University, Shanghai, China; ^2^ National Demonstration Center for Experimental Fisheries Science Education, Shanghai Ocean University, Shanghai, China; ^3^ Shanghai Engineering Research Center of Aquaculture, Shanghai Ocean University, Shanghai, China

**Keywords:** *elk1*, miR-462-731, macrophage, polarization, phagocytosis

## Abstract

MicroRNA clusters are microRNAs (miRNAs) that are distributed in close proximity on chromosomes. In this study, we report a miRNA cluster identified from grass carp (*Ctenopharyngodon idella*), miR-462-731, which plays a positive role in host antibacterial immunity. The expression of miR-462-731 was disrupted after infection by *Aeromonas hydrophila*. Transcription factor ETS transcription factor ELK1 was identified to bind to the promoter of the miR-462-731 cluster and suppress its expression. In addition, miR-731 negatively regulates the expression of *elk1*, forms an elk1/miR-462-731 double negative feedback loop. In addition, we found that miR-731 directly targets ezrin a (*ezra*), participates in inducing PI3K/AKT signaling in macrophage, to induce macrophage polarization to the M1 phenotype with stronger phagocytosis. Our results demonstrate a novel elk1/miR-462-731 feedback loop. The data deepen our understanding of the relationship between macrophage polarization and phagocytosis in teleost fish.

## Introduction

Studies have shown that miRNA genes tend to form clusters rather than being randomly distributed on chromosomes (1, 2). Genome duplication and *de novo* formation are important mechanisms for generating miRNA clusters (3). miRNAs in the same miRNA cluster may be transcribed in a polycistronic manner from common pri-miRNA transcripts, similar to the operon regulation systems in prokaryotes (3, 4). The consistency of the expression of miRNAs in miRNA clusters implies that they share common cis-regulatory elements, resulting in a cooperated function for those miRNAs (5). Furthermore, increasing evidence suggests that clustered miRNA genes are often co-expressed with neighboring miRNAs and host genes (6). Therefore, an increasing number of studies have reported that miRNAs in the same cluster can regulate functionally-related genes. For instance, overexpression of the miR-183-96-182 cluster suppresses the expression of zinc transporters (7); and the miR-23a~27a~24-2 cluster regulates mouse macrophage polarization (8).

miRNAs have emerged as powerful post-transcriptional regulators of gene expression (9), and it is estimated that over 30% of genes in the human genome are regulated by miRNAs (10). Studies showed that miRNAs regulate the differential expression of transcription factors in biological processes (11, 12). Transcription factors (TF) act as additional gene regulators that can bind to DNA and control transcriptional activation or repression (13, 14). Numerous studies have shown that TFs further regulate miRNA expression by binding to miRNA promoters (15–19). For example, transcription factor pu.1 activates miR-424 by binding to its promoter to encourage human macrophage differentiation (20). Notably, the mechanism of action of the feedback loops of the interactions between transcription factors and miRNAs are important for homeostasis (21, 22). Negative feedback control is a ubiquitous regulatory motif in many biological systems, essential for dynamic control in response to perturbations (23). Zebrafish possess the pu.1/miR-462-731 negative feedback loop, in which miR-731 negatively regulates the transcription factor PU.1, which in turn decreases the expression of miR-462-731 (24). However, there have been no studies on the regulatory mechanism of the miR-462-731 feedback loop in grass carp.

Macrophages are an important part of the immune system and play diverse roles during infection, inflammation, tissue damage, and repair (25, 26). Diversity and plasticity are two hallmarks of macrophages (27). During acute inflammation, macrophages exhibit an M1 activation state, including an enhanced ability to kill and phagocytose pathogens (28). Numerous studies have supported the notion that M1 macrophages have stronger phagocytosis (29). Phagocytosis is a critical cellular process for the induction of antimicrobial responses and regulation of adaptive immunity, and both teleost and mammalian macrophages show pro-inflammatory and homeostatic responses after phagocytosis (30). Phagocytosis in macrophages is regulated by LPS recognition receptors, such as TLR4 and CD14 (26). However, CD14 does not exist in teleost genome. Have study beloved to be *ezrin* was involved in the intracellular signal transduction, links TLR4 and PI3K/AKT signaling for induction in response to macrophages activation phagocytosis (31). Nevertheless, there have been few studies on the effect of miRNAs on phagocytosis after the regulation of macrophage polarization.

In a previous study, we identified 21 miRNAs significantly associated with antibacterial immune processes in spleen of grass carp that were susceptible and resistant to *Aeromonas hydrophila* (32). Additionally, higher expression of a miRNA cluster comprising miR-462 (33) and miR-731, only found in teleost, was observed in the spleen of resistant grass carp. In the current study, we aimed to explore the regulatory relationship between miR-462-731 and the transcription factor *elk1*, and discover whether the *elk1*/miR-462-731 feedback loop exists. Functional experiments demonstrated that miR-731 regulates macrophage polarization and phagocytosis.

## Materials and Methods

### Experimental Fish and Treatments

The experimental grass carp (average weight, 750 g) were obtained from Binhai Farm of Shanghai Ocean University, Shanghai, China. Before fish acclimation, the rearing tank was disinfected, the water in the tank was fully aerated, and suitable lighting conditions, 12 h light and 12 h dark, were provided for fish growth. The water temperature was maintained at 28 ± 2°C using a heater. The fish were fed for 2 weeks before the experiment. The fish were fed with 5% of their total body weight three times a day. The grass carp (n =180) were divided equally and randomly into six tanks, three tanks served as the control group, in which the fish were injected with 100 μL of phosphate buffered saline (PBS), while in the other tanks, the grass carp were injected into 100 μL of *A. hydrophila* (1 × 10^8^ colony forming units (CFU)/mL). Then, all tissues were randomly sampled at 4, 8, 12, 24, and 48 h and immediately frozen in liquid nitrogen at −80°C. All sampling tools were sterilized using 75% ethanol.

### Culture of *C. idella* Kidney Cells

The *C. idella* kidney cells (CIK) were provided by the China Center for Type Culture Collection (Wuhan, China). The cells were cultured in a 25 cm^2^ culture dishes with 6 mL of nutrient solution. The nutrient solution was M199 medium (Life Technologies, Carlsbad, CA, USA) supplemented with 10% heat-inactivated fetal bovine serum (FBS; Life Technologies) and 1% penicillin-streptomycin solution (100 ×) (Life Technologies). The cells were cultured at 28°C in a 5% CO_2_ incubator. Before the experiment, the CIK cells were adjusted to 2 × 10^6^ cells/mL final concentration and incubated in 6-well or 24-well plates for 24 h. All samplings and analyses were performed in triplicate. The cell samples were treated with 1 mL of TRIzol reagent (Invitrogen, Waltham, MA, USA) for RNA extraction.

### Grass Carp Macrophage Isolation and Primary Cell Culture

Fish livers were dissected out using sterilized scissors and tweezers. All tissues were washed three times in PBS containing 1% penicillin-streptomycin solution to eliminate impurities. Then, the tissues were homogenized using a syringe pushing head and filtered using a 70-mesh cell filter. The tissue filtrate was added dropwise to a 51% percoll solution to ensure that the interface was not broken. Centrifugation was performed at 4°C, 400g for 30 minutes. The white liquid in the middle layer was collected and re-suspended, centrifuged again at 4°C for 10 minutes, and the supernatant was discarded. The collected cells were seeded in the 6-well plates and cultured at 28°C for 6 h. Non-adherent cells were removed and adherent cells were incubated in complete medium (Dulbecco’s modified Eagle’s medium (DMEM), 10% FBS, 100 U/mL penicillin, 100 mg/mL streptomycin) at 28°C with 5% CO_2_.

### Sequence Analysis

The mature miRNA and miRNA precursors sequences of different species were obtained from the miRBase database (http://www.mirbase.org/). Alignment analysis of different species of mature miRNA sequences was performed using BioEdit software, and a phylogenetic tree derived from alignment of these miRNAs precursor sequences was constructed using the neighbor-joining (NJ) algorithm with bootstrapped 1000 times using the MEGA-X software (34). In addition, prediction of the secondary structure was carried out using the online tools RNAfold (35) and RNAalifold (36), to verify the hairpin structures of the precursors.

### Plasmid Construction

To construct an *elk1* expression plasmid, the full-length coding sequence of grass carp transcription factor *elk1* was amplified by PCR and inserted into vector pEGFP-N1 (Promega, Madison, WI, USA) to generate pEGFP-elk1. The sequence of the *elk1* promoter was inserted into vector pGL3-basic (Promega) to generate pGL3-pelk1. At the same time, the *elk1* and *ezra* fragment containing presumptive miR-731 target sequences were amplified by PCR. The amplicon was cloned into the dual luciferase vector pmirGLO (Promega) to generate pmirGLO-elk1*-*WT and pmirGLO-ezra*-*WT. Mutation of target sequences was performed using a MutanBEST Kit (Takara, Dalian, China) following standard procedures using the corresponding primers in **Table 1**. Pyrobest DNA Polymerase was used for PCR involving 30 cycles at 94°C for 30 s, 55°C for 30 s, and 72°C for 5 min. The resulting DNA fragment was blunted using Blunting Kination Enzyme Mix and ligated using ligation solution I at 16°C for 1 h. The ligation product was transformed into DH5a and all the constructed plasmids were confirmed by Sanger sequencing and were extracted using an Endotoxin-Free Plasmid DNA Miniprep Kit (Tiangen Biotech, Beijing, China) for further luciferase reporter assays. All primer sequences are listed in **Table 1**.

**Table 1:** Primers used in the present study.

Target	Forward primer (5’ - 3’)	Reverse primer (5’ - 3’)
miR-731	AATGACACGTTTTCTCCCGGATCG	10 x miScript Universal primer
miR-101a	TACAGTACTGTGATAACTGAAG	10 x miScript Universal primer
*ezra*	ACTGACAATGCCTAAGCCTATC	AGTACCAGACCTCACGCAAA
pmirGLO-ezra-WT	GCTAGCTGAATGGCAGAACAGGGCTA	TCTAGATGCTGGTGCTCGAGGTTTAC
pmirGLO-ezra-MUT	AATGTACTAGGACCAGGGGACAAGTACAAGACGCTTCG	CCCTGGTCCTAGTACATTTTCATTGTGCAGAATGTC
*elk1*	AGTGCCTGTCCCTGGAGTTA	GATGCCGTATTGGTGTCCTC
pmirGLO-elk1	GTGGTGTATTTCACTGTCATTC	CCTGTTCCAACCTCTGGT
pEGFP-elk1	GCTAGCATGGAGTCCAACCCGCTGAT	GAGCTCTCAAGGTTTCTGTGGTCCAG
pGL3-pelk1	GAGCTCCCTCTTGTTCATCACAGTGACA	GCTAGCAGAAGGAGAAGGGGGGGTTA
*il1b*	TGAAGTCTGTGATTCGGCTA	TTGAAGGAGGTCACTGAAAC
*tnfa*	ACCCTGAAGTCTCTAATAAAACCC	GTGGCTCATATGCACAATGTCT
*il10*	ACGAGAACGTGCAACAGA	TGGCAAACTCAAAGGGAT
*tgfb*	TTACGGCTTCGGATTAAG	TGGCAGTGTCACCTCTCT
18s *rRNA*	GGACACGGAAAGGATTGACAG	CGGAGTCTCGTTCGTTATCGG

### RNA Extraction and Quantitative Real-Time Reverse Transcription PCR

Total RNA was extracted using the *TRIzol* reagent. The concentration was measured using a Nanodrop 2000 instrument (Thermo Fisher Scientific, Waltham, MA, USA) and the RNA integrity was visualized using agarose gel electrophoresis. Then, 1 μg of the total RNA was reverse transcribed using an Evo M-MLV RT Kit with gDNA Clean for qPCR (Accurate Biotechnology, Hunan, China). For miRNA, cDNA was prepared using a miScript II RT Kit (Qiagen, Germany). The qPCR step was conducted on a CFX96 instrument (Bio-Rad Laboratories, Hercules, CA, USA) using a SYBR Green Premix Pro Taq HS qPCR Kit (Accurate Biotechnology) to quantify the expression of *ezra*. qPCR was performed in a final volume of 25 μL and each reaction included 9.5 μL ddH_2_O, 12.5 μL of 2 × SYBR Green Pro Taq HS Premix, 0.5 μL forward primer (10 μM), 0.5 μL reverse primer (10 μM), and 2 μL of the prepared cDNA. The following qPCR cycling conditions were used: 1 cycle at 95°C for 10 s; followed by 40 cycles at 95°C for 5 s, 60°C for 20 s, and dissociation curve analysis was performed after each assay to determine target specificity. miR-101 for miRNA and 18S *rRNA* for mRNA were used to normalize the relative expression of miRNA (37) and mRNA, respectively. The primers used for qPCR are listed in **Table 1**. Each experimental group was analyzed in quadruplicate.

### Transfection and Dual Luciferase Reporter Detection

For the transfection experiment, CIK cells from 24-well plates were cultured in M199 medium containing 10% FBS and 1% penicillin-streptomycin solution with 5% CO_2_ at 28°C. After 24h, pmirGLO-ezra or empty vector were transfected into CIK cells simultaneously with 100 nM miR-731 agomir or miR-731 antagomir (GenePharma, Shanghai, China) or the negative control (NC) using the Lipofectamine 3000™ (Invitrogen) transfection reagent. pEGFP-elk1 or pEGFP-N1 empty plasmid were cotransfection with pGL3-pelk1 into CIK cells. At 24h or 48h after transfection, firefly and Renilla luciferase activities were measured using a dual luciferase reporter assay (Promega). Firefly luciferase activity was normalized to Renilla luciferase activity.

### Fluorescein Isothiocyanate Fluorescent Labeling of *A. hydrophila*

An overnight culture of *A. hydrophila* was washed three times with PBS. Then, 0.1 M Na_2_CO_3_ was added together with FITC dissolved in PBS (1 mg/mL). The tube containing the cells was wrapped in foil, fixed to the turntable machine, and rotated for 30 min. Finally, the cells were washed with PBS until the supernatant showed no color.

### Phagocytosis Assay

After the isolated macrophages were cultured for 6 h, they were washed three times with PBS and fresh medium was added. In addition, the miR-731 agomir, antagomir, and NC were transfected into macrophages. After 5 h, FITC-labeled *A. hydrophila* (2 × 10^9^ CFU/mL) were infected into macrophages for 30 min. Then, cell scrapers were used to scrape off macrophages, and prepare a single cell suspension. All sample data were the acquired on an ImageStream^®^X Mark II (Luminex Corp., Austin, TX, USA) using a 495 and 519 nm laser. A total of 10000 events were collected for each sample.

### Statistical Analysis

Gene expression data were obtained and calculated using the 2^−ΔΔCT^ method. The data among different groups were analyzed using one-way analysis of variance (ANOVA) followed by Duncan multiple comparison test. All data were presented as the mean value ± SD. Significant differences between each two groups were determined using a two-tailed Student’s t-test. *p < 0.05, **p < 0.01, ***p < 0.001 indicated statistical significance.

## Results

### Characteristic Analysis of miR-462-731 and Orthologs miR-191-425

**Figure 1:**
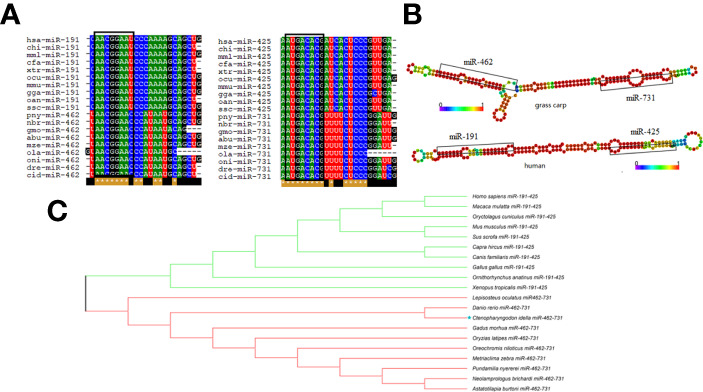
Evolutionary analysis of miR-462-731. **(A)** Alignment analysis of different species of mature miRNA sequences was performed using BioEdit software. **(B)** Analysis of the precursor structures of miR-191-425 cluster and miR-462-731 cluster. **(C)** phylogenetic tree derived from alignment of miR-191-425 and miR-462-731 sequences was constructed using the neighbor-joining (NJ) algorithm with bootstrapped 1000 times using the MEGA-X software.

Alignment of the mature sequences of miR-462-731 and miR-191-425 from different species revealed that the mature sequences shared a high conservation region that corresponded to the seed sequences (**Figure 1A**). RNAfold and RNAalifold prediction of the secondary structure showed that the precursor sequences of miR-462-731 and miR-191-425 have diverse structures (**Figure 1B**). A phylogenetic tree derived from these alignments indicated that teleost miR-462-731 grouped with mammalian miR-191-425, indicating a close genetic relationship in the evolutionary chronogram (**Figure 1C**). Compared with that of the pre-miR-462-731 sequence, the seed sequence is highly conserved.

### *elk1*/miR-462-731 Negative Feedback Loop

**Figure 2:**
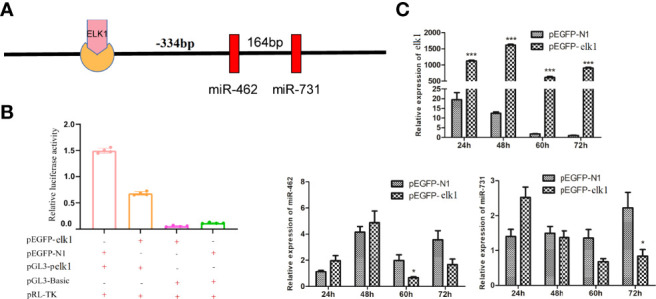
Transcription factor ELK1 can bind to the miR-462-731 cluster promoter fragment and regulate the expression of miR-462-731. **(A)** Predicted binding site of ELK1 to the promoter fragment of the miR-462-731 cluster. **(B)** CIK cells were transfected with the recombinant plasmids pEGFP-elk1 or pEGFP-N1, along with pGL3-pelk1 for 48 h, and the luciferase activity was determined. **(C)** Expression profiles of elk1 miR-462 and miR-731 in *C.Idella* Kidney cells (CIK) cells at 24, 48, 60 and 72 h following transfected pEGFP-elk1 or pEGFP-N1. All values represent the mean ± SD of three independent experiments. Asterisks indicate significant differences (*p < 0.05, ***p < 0.001).

Previous studies confirmed that miR-462-731 is a specific miRNA cluster in teleost fish. To better understand the regulatory mechanism of miR-462-731 cluster, the transcription factor elk1 binding sites on the miR-462-731 promoter ( −334 to -340 bp) were predicted (**Figure 2A**). To confirm this analysis, we constructed a luciferase reporter plasmid for the upstream promoter of miR-462-731 and an overexpression vector for *elk1* (pEGFP-elk1). As shown in **Figure 2B**, lower luciferase activity was produced by the luciferase reporter pGL3-pelk1 + pEGFP-elk1 compared with that achieved with pGL3-pelk1 + pEGFP-N1 empty plasmid. The results showed that overexpression of *elk1* inhibited the expression of miR-462-731 (**Figure 2C**).

**Figure 3:**
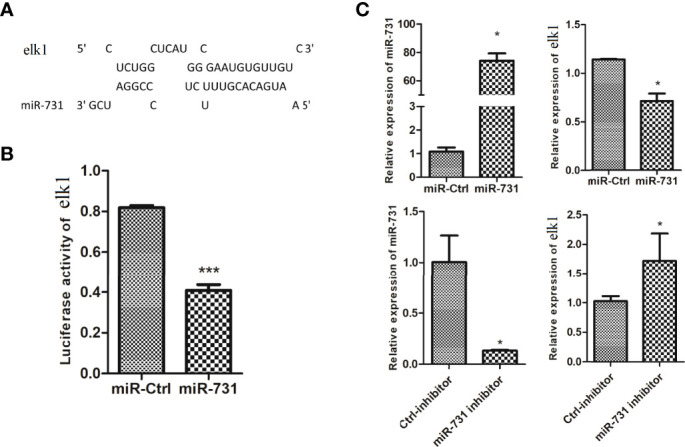
Prediction and validation of the relationship of *elk1* and miR-731. **(A)** Binding site of miR-731 and *elk1*. **(B, C)** *C. idella* Kidney cells (CIK) were transfected with miR-731 agomir or control solution, along with the recombinant plasmids pmirGLO-elk1 for 24 h, and the luciferase activity was determined. All values represent the mean ± SD of three independent experiments. Asterisks indicate significant differences (*p < 0.05, ***p < 0.001).

On the other hand, *elk1* was identified as a potential target of miR-731, with a complementary binding site on *elk1* according to miRanda and Targetscan (**Figure 3A**). First, an miR-731 agomir was co-transfected with the construct pmirGLO-elk1 into CIK cells, with NC as a control. At 24 h after transfection, the cells were harvested and assayed for luciferase activity and normalized to Renilla luciferase activity. Overexpression of miR-731 significantly reduced the relative luciferase activity (**Figure 3B**). Furthermore, when the miR-731 agomir and antagomir were transfected into CIK cells, the expression of miR-731 was increased and decreased, respectively. The expression of *elk1* was decreased and increased after transfection with the miR-731 agomir and antagomir, respectively (**Figure 3C**).

### *Ezra* Is a Target Gene of miR-731

**Figure 4:**
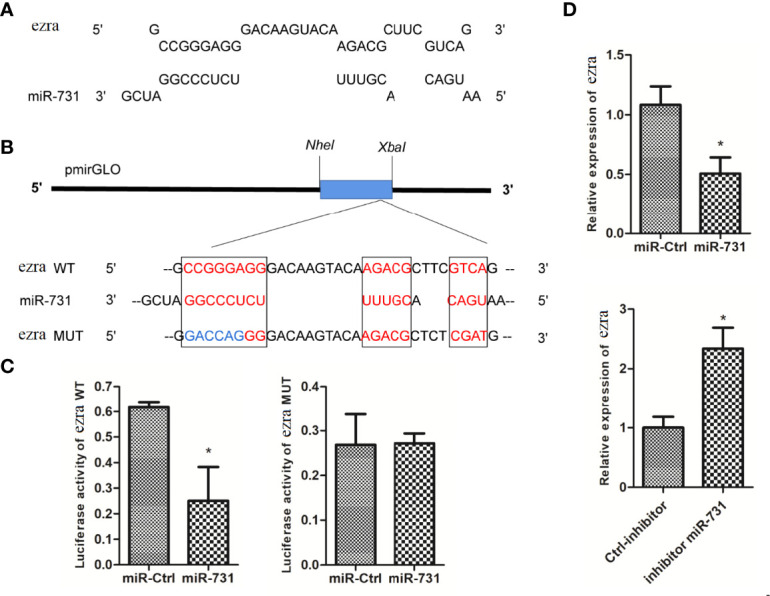
Prediction and validation of target genes of miR-731. **(A)** Binding site of miR-731 and *ezra*. **(B)** Schematic diagram of the NheI/Xbal sites. **(C, D)** *C. idella* Kidney cells (CIK) were transfected with miR-731 agomir or control solution, along with the recombinant plasmids pmirGLO-ezra-WT or pmirGLO-ezra-MUT for 24 h, and the luciferase activity was determined. All values represent the mean ± SD of three independent experiments. Asterisks indicate significant differences (*p < 0.05).

The miRanda and Targetscan programs predicted *ezra* as a target gene of miR-731 (**Figure 4A**). The *ezra* reporter plasmid (pmirGLO-ezra-WT) was constructed to investigate the interaction between miR-731 and *ezra*, and the mutation vector at the miR-731 binding site was used as the control (pmirGLO-ezra-MUT) (**Figure 4B**).

After transfection of the miR-731 agomir, the luciferase activity of pmirGLO-ezra-WT was significantly inhibited, whereas both the control and miR-731 agomir showed no effect on the activity of the luciferase reporter containing the pmirGLO-ezra-MUT (**Figure 4C**). The expression of *elk1* was decreased after transfecting the miR-731 agomir and increased after antagomir transfection (**Figure 4D**). These results showed that miR-731 directly targets *ezra* in grass carp.

### The Expression of miR-731 and *ezra* Was Disordered Upon *A. hydrophila* Infection

**Figure 5:**
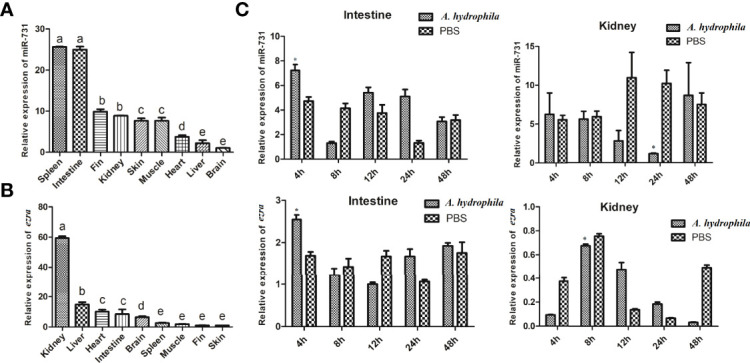
Analysis of miR-731 and *ezra* expression by qRT-PCR. **(A, B)** in 9 tissues of grass carp. **(C)** in grass carp intestine and kidney 4, 8, 12, 24 and 48 h after *A. hydrophila* infection. All values represent the mean ± SD of three independent experiments. Different lowercase letters indicate statistically significant differences (p < 0.05), asterisks indicate significant differences (*p < 0.05).

In this study, we found miR-731 was constitutive expressed in all tested tissues, and was significantly and highly expressed in the spleen and intestine *in vivo* (**Figure 5A**), while *ezra* was significantly and highly expressed in the kidney (**Figure 5B**). Furthermore, the expression levels of miR-731 and *ezra* were determined at various time points after *A. hydrophila* stimulation. Compared with that in the PBS-injected grass carp, the expression levels of miR-731 and *ezra* in grass carp kidney and intestines fluctuated after *A. hydrophila* (10^8^ CFU/mL) infection (**Figure 5C**).

### miR-731 Promotes Phagocytosis of Macrophages and the Expression of Inflammatory Cytokines

**Figure 6:**
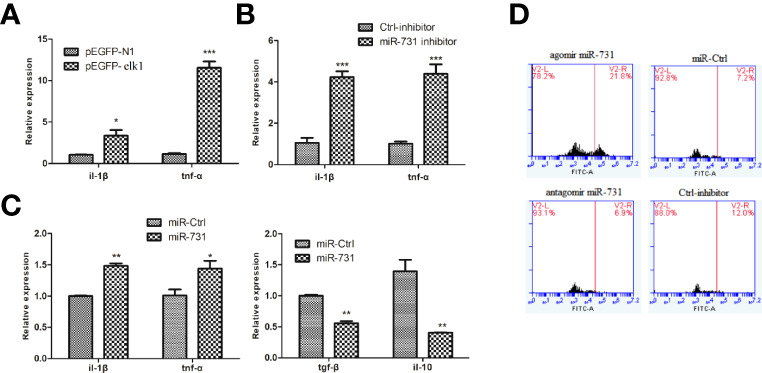
*C. idella* Kidney cells (CIK) were transfected with either the pEGFP-elk1 **(A)** or miR-731 antagomir **(B)**. After 48 h, the level of *il1b* and *tnfa* expression was determined using qPCR. Macrophages were transfected with miR-731 agomir or antiagomir or control solution, and after 5h the macrophages were infected with *A. hydrophila*. The mRNA levels of *il1b*, *tnfa*, *il10*, and *tgfb* **(C)** were analyzed by qRT-PCR 0.5 h after infection. **(D)** FITC fluorescence in the macrophages were analyzed by the ImageStream imaging-flow platform. All values represent the mean ± SD of three independent experiments. Asterisks indicate significant differences (*p < 0.05, **p < 0.01, ***p < 0.001).

In CIK cells, the mRNA expression levels of downstream inflammatory factors, *tnfa* and *il1b*, were significantly upregulated in the *elk1* overexpression group (**Figure 6A**) and in the miR-731 inhibition group (**Figure 6B**). Moreover, to investigate the effect of miR-731 on the response of *A. hydrophila-*stimulated macrophages, miR-731 agomir/antagomir or NC were transfected into macrophages for 5 h, which were then infected with *A. hydrophila* for 30 min. We found increased expression of pro-inflammatory cytokines (*tnfa* and *il1b*), but reduced expression of anti-inflammatory cytokines (*tgfb* and *il10*) (**Figure 6C**).

To assess phagocytosis, macrophages were exposed to FITC-labeled *A. hydrophila* for 30 min. The ImageStream imaging-flow platform showed increased FITC fluorescence in the macrophages transfected with the miR-731 agomir compared with that in the control, but decreased FITC fluorescence in the macrophages transfected with the miR-731 antagomir (**Figure 6D**). These results suggested that miR-731 promoted phagocytosis of *A. hydrophila via* macrophages during the defense against bacterial infection.

## Discussion

The miR-462-731 cluster has only been described in teleost fishes but is highly conserved among them. Studies showed that the teleost miR-462-731 cluster ortholog, the miR-191-425 cluster, is found in human and mammals, and in a more primitive cartilaginous fish, elephant shark (*Callorhinchus milii*) (38, 39). Despite the miR-462-731 cluster and miR-191-425 cluster being highly conserved, the differences in the expression regulation indicate that they are functionally diverse in different vertebrates (38). In teleost, the miR-462-731 cluster is mainly involved in hypoxia and immune responses. In addition, studies have shown that the interaction between miRNAs and TFs can form a negative feedback loop. In this study, we focused on the evolution of the miR-462-731 cluster, the negative feedback loop of elk1/miR-462-731, and the function of miR-731 in the immune response of grass carp infected with *A. hydrophila*.

Although the seed sequences of miR-462-731 and miR-191-425 are highly conserved, their target genes and function are different. Studies have demonstrated that the miR-191-425 cluster is involved in humans cell proliferation and tumorigenesis (40, 41). In zebrafish, the miR-462-731 cluster not only inhibits cell proliferation, but also participates in the regulation of the hypoxia response (42). In addition, the miR-462-731 cluster found in rainbow trout (*Oncorhynchus mykiss*) can act as a key switch to suppress the antiviral immune response in teleost. In this study, the stem-loop structure in the secondary structure of teleost miR-462-731 and mammalian miR-191-425 were shown to be quite different. The secondary structure of miRNA can affect the processing of mature miRNAs (43), and can alter the regulation of target genes (44), further illustrating the functional differential evolution of miR-462-731 and miR-191-425 regulation. Earlier analyses showed that conservation of miRNA sequences between distantly related species might not necessarily imply functional conservation, especially between species with larger physiological differences (45). This was further illustrated by our findings that the miR-462-731 cluster has generated new functions during evolution.

Positive feedback loops and double negative feedback loops in cell signaling systems act as bistable switches that directly determine cell states (46, 47). miRNAs and their target transcription factors mainly ensure the stability of the state through interactions in double negative feedback loops (48), two switches in a double negative feedback loop counterbalance each other. For example, the miR-200-Zeb1 double-negative feedback loop is reversible, the transcription factor *zeb1* represses the transcription of miR-200, which in turn targets and represses *zeb1* expression (49). Genome-scale TF-miRNA regulatory network studies revealed not only the existence of reciprocal regulation between miRNAs and TFs, but also TFs and miRNAs coordinate regulation of gene expression (22). Our results showed that there is a double negative feedback loop between miR-462-731 and *elk1* in grass carp. Gene overexpression and dual luciferase reporter assays suggested that *elk1* repressed the miR-462-731 cluster promoter and decreased the miR-462-731 expression level in CIK cells. Besides, we also identified that miR731 negatively regulates *elk1* expression. ELK1 is a member of the ETS domain family of transcription factors (50), which are implicated in regulating cell migration, inflammation, and the immune response (51). The overexpression of *elk1* in macrophages significantly inhibited the phagocytosis of macrophages on cancer cells (52). More recently, studies showed that miR-462 and miR-731 could regulate inflammation and apoptosis in grass carp (33, 53). In addition, studies have demonstrated that the transcription factor c-Myc regulates the expression of *E2F1* by regulating the transcription of the miR-17-92 cluster (54). Herein, we found that overexpression *elk1* and inhibition miR-731 could upregulate the expression of *tnfa* and *il1b*. However, further research is needed on the mechanism of the elk1/miR-462-731 feedback loop in fish immunity.

We have verified that miR-731 can directly target and regulate *ezra*. In addition, the expression of *ezra* was also affected by *elk1*. EZRA, a member of the ezrin/moesin/radixin (ERM) family, is involved in signal transduction. *ezrin* directly links cystic fibrosis transmembrane conductance regulator (CFTR) to phosphatidylinositol-4,5-bisphosphate 3-kinase (PI3K)/protein kinase B (AKT) signaling, and loss of *ezrin* results in compromised PI3K/AKT signaling that promotes macrophage resistance to bacterial invasion (31). Macrophages are a heterogeneous cell population (55), whose main functional variability depends on two polarization states. M1 macrophages show a strong phagocytic activity to eliminate bacteria and damaged cells, and have been shown to have anti-tumorigenic properties, while M2 macrophages exhibit a phenotype roughly opposite to that of M1 macrophages (56–59). Overexpression of miR-451a enhanced the phagocytic capacity of macrophages, and miR-451a also increased the proliferative capacity of M1 and M2 polarized macrophages (60). In this study, the levels of inflammatory factors (*il1b*, *tnfa*, *il10*, and *tgfb*) showed that miR-731 overexpression promotes M1 polarization, and the increased FITC fluorescence in the macrophages showed that M1 macrophages have a stronger phagocytic ability for *A. hydrophila*. Overall, these findings expanded our understanding of the effect of miRNAs on macrophages and the correlation between macrophage polarization and phagocytosis in teleost fish.

In summary, the results showed that the transcription factor *elk1* represses the expression of the miR-462-731 cluster, and that miR-731 negatively regulates *elk1* expression, forming a double negatively feedback loop. miR-731 targeting *ezra* promotes polarization into M1 macrophages and stronger phagocytosis of *A. hydrophila*. This observation suggests that the miR-462-731 cluster plays an important role in promoting the antibacterial immune response of teleost fish. However, the precise regulatory mechanism between miR-731 and the transcription factor *elk1* requires further study.

## Data Availability Statement

The original contributions presented in the study are included in the article/supplementary material. Further inquiries can be directed to the corresponding authors.

## Ethics Statement

The animal study was reviewed and approved by Institutional Animal Care and Use Committee (IACUC).

## Author Contributions

YH, XYX and JLL contributed to conceive and design the experiments. YH performed the majority of the experiments, with the help of YTL, YYY, YL and XWJ. YH, and XYX analyzed the data. YH drafted the manuscript. All authors have read and agreed to the published version of the manuscript.

## Funding

This work was supported by the National Natural Science Foundation of China Youth Project (grant number 31802285), and China’s Agricultural Research System (CARS-45-03) and Training plan for applied talents integrating industry and education-Collage of Future Technology.

## Conflict of Interest

The authors declare that the research was conducted in the absence of any commercial or financial relationships that could be construed as a potential conflict of interest.

## Publisher’s Note

All claims expressed in this article are solely those of the authors and do not necessarily represent those of their affiliated organizations, or those of the publisher, the editors and the reviewers. Any product that may be evaluated in this article, or claim that may be made by its manufacturer, is not guaranteed or endorsed by the publisher.

## References

[1] Lai EC Tomancak P Williams RW Rubin GM. Computational Identification of Drosophila microRNA Genes. Genome Biol (2003) 4:R42. doi: 10.1186/gb-2003-4-7-r42 12844358 PMC193629

[2] Zhang YF Zhang R Su B. Diversity and Evolution of MicroRNA Gene Clusters. Sci China (2009) 52:261. doi: 10.1007/s11427-009-0032-5 19294351

[3] Wang Y Luo J Hong Z Jian L. microRNAs in the Same Clusters Evolve to Coordinately Regulate Functionally Related Genes. Mol Biol Evol (2016) 33:2232–47. doi: 10.1093/molbev/msw089 PMC4989102 27189568

[4] Ghildiyal M Zamore PD. Small Silencing RNAs: An Expanding Universe. Nat Rev Genet (2009) 10:94–108. doi: 10.1038/nrg2504 19148191 PMC2724769

[5] Yu J Wang F Yang GH Wang FL Ma YN Du ZW. Human microRNA Clusters: Genomic Organization and Expression Profile in Leukemia Cell Lines. Biochem Bioph Res Co (2006) 349:59–68. doi: 10.1016/j.bbrc.2006.07.207 16934749

[6] Baskerville S Bartel DP. Microarray Profiling of microRNAs Reveals Frequent Coexpression With Neighboring miRNAs and Host Genes. RNA (2005) 11:241–7. doi: 10.1261/rna.7240905 PMC1370713 15701730

[7] Mihelich BL Khramtsova EA Arva N Vaishnav A Johnson DN Giangreco AA. miR-183-96-182 Cluster Is Overexpressed in Prostate Tissue and Regulates Zinc Homeostasis in Prostate Cells. J Biol Chem (2011) 286:44503–11. doi: 10.1074/jbc.M111.262915 PMC3247959 22045813

[8] Boucher A Klopfenstein N Hallas WM Skibbe J Appert A Jang SH. The miR-23a∼27a∼24-2 microRNA Cluster Promotes Inflammatory Polarization of Macrophages. J Immunol (2021) 206:540–53. doi: 10.4049/jimmunol.1901277 PMC7855803 33328213

[9] Carthew RW Sontheimer EJ. Origins and Mechanisms of miRNAs and siRNAs. Cell (2022) 136:642–55. doi: 10.1016/j.cell.2009.01.035 PMC2675692 19239886

[10] Lewis BP Burge CB Ba Rtel DP. Conserved Seed Pairing, Often Flanked by Adenosines, Indicates That Thousands of Human Genes are microRNA Targets. Cell (2005) 120:15. doi: 10.1016/j.cell.2004.12.035 15652477

[11] Johnson SM Lin SY Slack FJ. The Time of Appearance of the C. Elegans Let-7 microRNA Is Transcriptionally Controlled Utilizing a Temporal Regulatory Element in Its Promoter. Dev Biol (2003) 259:364–79. doi: 10.1016/S0012-1606(03)00202-1 12871707

[12] Martinez NJ Ow MC Reece-Hoyes JS Barrasa MI Ambros VR Walhout A. Genome-Scale Spatiotemporal Analysis of Caenorhabditis Elegans microRNA Promoter Activity. Genome Res (2008) 18:2005–15. doi: 10.1101/gr.083055.108 PMC2593583 18981266

[13] Sharrocks AD Brown AL Ling Y Yates PR. The ETS-Domain Transcription Factor Family. Int J Biochem Cell Biol (1997) 29:1371–87. doi: 10.1038/35099076 9570133

[14] Yang SH Shore P Willingham N Lakey JH Sharrocks AD. The Mechanism of Phosphorylation-Inducible Activation of the ETS-Domain Transcription Factor Elk-1. EMBO J (1999) 18:5666–74. doi: 10.1093/emboj/18.20.5666 PMC1171633 10523309

[15] Guo Q Wang T Yang Y Gao L Zhao Q Zhang W. Transcriptional Factor Yin Yang 1 Promotes the Stemness of Breast Cancer Cells by Suppressing miR-873-5p Transcriptional Activity. Mol Ther- Nucl Acids (2020) 21:527–41. doi: 10.1016/j.omtn.2020.06.018 PMC7381513 32711380

[16] Hamurcu Z Sener EF Taheri S Nalbantoglu U Kokcu ND Tahtasakal R. MicroRNA Profiling Identifies Forkhead Box Transcription Factor M1 (FOXM1) Regulated miR-186 and miR-200b Alterations in Triple Negative Breast Cancer. Cell Signal (2021) 83:109979. doi: 10.1016/j.cellsig.2021.109979 33744419

[17] Qian S Wang W Li M. Transcriptional Factor Yin Yang 1 Facilitates the Stemness of Ovarian Cancer *via* Suppressing miR-99a Activity Through Enhancing Its Deacetylation Level. BioMed Pharmacother (2020) 126:110085. doi: 10.1016/j.biopha.2020.110085 32199224

[18] Chen C Xiang H Peng Y Peng J Jiang S. Mature miR-183, Negatively Regulated by Transcription Factor GATA3, Promotes 3T3-L1 Adipogenesis Through Inhibition of the Canonical Wnt/β-Catenin Signaling Pathway by Targeting LRP6. Cell Signal (2014) 26:1155–65. doi: 10.1016/j.cellsig.2014.02.003 24556500

[19] Wang H Luo J He Q Yao D Wu J Loor JJ. miR-26b Promoter Analysis Reveals Regulatory Mechanisms by Lipid-Related Transcription Factors in Goat Mammary Epithelial Cells. J Dairy Sci (2017) 100:5837–49. doi: 10.3168/jds.2016-12440 28527797

[20] Rosa A Ballarino M Sorrentino A Sthandier O Angelis F Marchioni M. The Interplay Between the Master Transcription Factor PU.1 and miR-424 Regulates Human Monocyte/Macrophage Differentiation. P Natl Acad Sci USA (2007) 104:19849–54. doi: 10.1073/pnas.0706963104 PMC2148386 18056638

[21] Alidadiania N Ghaderia S Nafi Dilaver. Alidadiani N Ghaderi S Dilaver N Bakhshamin S. Epithelial Mesenchymal Transition Transcription Factor (TF): The Structure, Function and microRNA Feedback Loop. Gene (2018) 674:115–20. doi: 10.1016/j.gene.2018.06.049 29936265

[22] Martinez NJ Walhout AJM. The Interplay Between Transcription Factors and microRNAs in Genome-Scale Regulatory Networks. Bioessays (2010) 31:435–45. doi: 10.1002/bies.200800212 PMC3118512 19274664

[23] Fagerlund R Behar M. Fortmann KT. Anatomy of a Negative Feedback Loop: The Case of Iκbα. J R Soc Interface (2015) 12:20150262–20150262. doi: 10.1098/rsif.2015.0262 26311312 PMC4614452

[24] Huang C-X Huang Y Duan X-K Zhang M Tu J-P Liu J-X. Zebrafish miR-462-731 Regulates Hematopoietic Specification and Pu.1-Dependent Primitive Myelopoiesis. Cell Death Differ (2019) 26:1531–44. doi: 10.1038/s41418-018-0234-0 PMC6748110 30459392

[25] Okabe Y Medzhitov R. Tissue Biology Perspective on Macrophages. Nat Immunol (2016) 17:9–17. doi: 10.1038/ni.3320 26681457

[26] Lu X-J Chen J. Specific Function and Modulation of Teleost Monocytes/Macrophages: Polarization and Phagocytosis. Zool Res (2019) 40:146–50. doi: 10.24272/j.issn.2095-8137.2019.035 PMC6591158 31011129

[27] Liu Y-C Zou X-B Chai Y-F Yao Y-M. Macrophage Polarization in Inflammatory Diseases. Int J Biol Sci (2014) 10:520–9. doi: 10.7150/ijbs.8879 PMC4046879 24910531

[28] Biswas SK Mantovani A. Macrophage Plasticity and Interaction With Lymphocyte Subsets: Cancer as a Paradigm. Nat Immunol (2010) 11:889–96. doi: 10.1038/ni.1937 20856220

[29] Deng H Li Z Tan Y Guo Z Zhi F. A Novel Strain of Bacteroides Fragilis Enhances Phagocytosis and Polarises M1 Macrophages. Sci Rep-UK (2016) 6:29401. doi: 10.1038/srep29401 PMC4933912 27381366

[30] Rieger AM Konowalchuk JD Leon G Katzenback BA Havixbeck JJ Kiemele MD. Fish and Mammalian Phagocytes Differentially Regulate Pro-Inflammatory and Homeostatic Responses. In Vivo PloS One (2012) 7:e47070. doi: 10.1371/journal.pone.0047070 23110059 PMC3479104

[31] Pietro CD Zhang PX O’Rourke TK Murray TS Wang L Britto CJ. Ezrin Links CFTR to TLR4 Signaling to Orchestrate Anti-Bacterial Immune Response in Macrophages. Sci Rep-UK (2017) 7:10882. doi: 10.1038/s41598-017-11012-7 PMC5589856 28883468

[32] Xu X Shen Y Fu J Lu L Li J. Next-Generation Sequencing Identified microRNAs That Associate With Motile Aeromonad Septicemia in Grass Carp. Fish Shellfish Immun (2015) 45:94–103. doi: 10.1016/j.fsi.2015.02.008 25698074

[33] Wang A Tao L Zhou F Xu X Shen Y Li J. miR-462 Modulates Cellular Immune Response by Targeting Cx32.2, Slc9a3.1 and Tbk1 in CIK Cells Infected With. Aeromonas Hydrophila Chin J Fisheries (2017) 43:1–11. doi: 10.1007/s10695-017-0341-8

[34] Kumar S Stecher G Li M Knyaz C Tamura K. MEGA X: Molecular Evolutionary Genetics Analysis Across Computing Platforms. Mol Biol Evol (2018) 35(6):1547–9. doi: 10.1093/molbev/msy096 PMC5967553 29722887

[35] Hofacker Priwitzer Stadler. Prediction of Locally Stable RNA Secondary Structures for Genome-Wide Surveys. Bioinformatics (2004) 20:186–90. doi: 10.1093/bioinformatics/btg388 14734309

[36] Hofacker IL Stadler PF. Automatic Detection of Conserved Base Pairing Patterns in RNA Virus Genomes. Comput Chem (1999) 23:401–14. doi: 10.1016/S0097-8485(99)00013-3 10404627

[37] Xu XY Shen YB Fu JJ Lu LQ Li JL. Determination of Reference microRNAs for Relative Quantification in Grass Carp (*Ctenopharyngodon Idella*). Fish Shellfish Immun (2014) 36:374–82. doi: 10.1016/j.fsi.2013.12.007 24368222

[38] Schyth BD Bela-ong DB Jalali SAH Kristensen LBJ Einer-Jensen K Pedersen FS. Two Virus-Induced MicroRNAs Known Only From Teleost Fishes Are Orthologues of MicroRNAs Involved in Cell Cycle Control in Humans. PloS One (2015) 10:e0132434. doi: 10.1371/journal.pone.0132434 26207374 PMC4514678

[39] Venkatesh B Lee AP Ravi V Maurya AK Lian MM Swann JB. Elephant Shark Genome Provides Unique Insights Into Gnathostome Evolution. Nature (2014) 505:174–9. doi: 10.1038/nature12826 PMC3964593 24402279

[40] Zhang X Wu M Chong Q-Y Weijie Z Pengxu Q Hong Y. Amplification of Hsa-miR-191/425 Locus Promotes Breast Cancer Proliferation and Metastasis by Targeting Dicer1. Carcinogenesis (2018) 39:1506–16. doi: 10.1093/carcin/bgy102 30084985

[41] Leva GD Piovan C Gasparini P Ngankeu A Taccioli C Briskin D. Estrogen Mediated-Activation of miR-191/425 Cluster Modulates Tumorigenicity of Breast Cancer Cells Depending on Estrogen Receptor Status. PloS Genet (2013) 9:e1003311. doi: 10.1371/journal.pgen.1003311 23505378 PMC3591271

[42] Huang C-X Chen N Wu X-J. The Zebrafish miR-462/miR-731 Cluster Is Induced Under Hypoxic Stress *via* Hypoxia-Inducible Factor 1α and Functions in Cellular Adaptations. FASEB J (2015) 29:4901–13. doi: 10.1096/fj.14-267104 26265472

[43] Mateos JL Bologna NG Chorostecki U Palatnik JF. Identification of microRNA Processing Determinants by Random Mutagenesis of Arabidopsis MIR172a Precursor. Curr Biol (2010) 20:49–54. doi: 10.1016/j.cub.2009.10.072 20005105

[44] Du Q Meng Z Wei G Sun S Li WX. microRNA/microRNA* Complementarity Is Important for the Regulation Pattern of NFYA5 by Mir169 Under Dehydration Shock in Arabidopsis. Plant J (2017) 91:22. doi: 10.1111/tpj.13540 28332758

[45] Ason B Darnell DK Wittbrodt B Berezikov E Kloosterman WP Wittbrodt J. Differences in Vertebrate microRNA Expression. PNAS (2006) 103:14385–9. doi: 10.1073/pnas.0603529103 PMC1599972 16983084

[46] Ferrell J Jr. Self-Perpetuating States in Signal Transduction: Positive Feedback, Double-Negative Feedback and Bistability. Curr Opin Cell Biol (2002) 14:140–8. doi: 10.1016/s0955-0674(02)00314-9 11891111

[47] Ferrell JE Xiong W. Bistability in Cell Signaling: How to Make Continuous Processes Discontinuous, and Reversible Processes Irreversible. Chaos (2001) 11:227–36. doi: 10.1063/1.1349894 12779456

[48] Tsang J Zhu J van Oudenaarden A. MicroRNA-Mediated Feedback and Feedforward Loops Are Recurrent Network Motifs in Mammals. Mol Cell (2007) 26:753–67. doi: 10.1016/j.molcel.2007.05.018 PMC2072999 17560377

[49] Title AC Silva PN Godbersen S Hasenöhrl L Stoffel M. The miR-200–Zeb1 Axis Regulates Key Aspects of β-Cell Function and Survival *In Vivo* . Mol Metab (2021) 53:101267. doi: 10.1016/j.molmet.2021.101267 34116231 PMC8258987

[50] Yordy JS Muise-Helmericks RC. Signal Transduction and the Ets Family of Transcription Factors. Oncogene (2000) 19:6503. doi: 10.1038/sj.onc.1204036 11175366

[51] Xu Z Dziarski R Wang Q Swartz K Sakamoto KM Gupta D. Bacterial Peptidoglycan-Induced Tnf-α Transcription Is Mediated Through the Transcription Factors Egr-1, Elk-1, and NF-κb. J Immunol (2001) 167:6975–82. doi: 10.4049/jimmunol.167.12.6975 11739517

[52] Wang X Luo X Chen C Tang Y Li L Mo B. The Ap-2α/Elk-1 Axis Regulates Sirpα-Dependent Tumor Phagocytosis by Tumor-Associated Macrophages in Colorectal Cancer. Sig Transduct Target Ther (2020) 5:1–12. doi: 10.1038/s41392-020-0124-z PMC7156469 32296015

[53] Fang Y Xu X-Y Tao L Shen Y Li J. Effects of microRNA-731 on Inflammation and Apoptosis by Targeting CiGadd45aa in Grass Carp. Fish Shellfish Immun (2020) 97:493–9. doi: 10.1016/j.fsi.2019.12.029 31838144

[54] O’Donnell KA Wentzel EA Zeller KI Dang CV Mendell JT. C-Myc-Regulated microRNAs Modulate E2F1 Expression. Nature (2005) 435:839–43. doi: 10.1038/nature03677 15944709

[55] Gordon S Taylor PR. Monocyte and Macrophage Heterogeneity. Nat Rev Immunol (2005) 5:953–64. doi: 10.1038/nri1733 16322748

[56] Grivennikov SI Greten FR Karin M. Immunity, Inflammation, and Cancer. Cell (2010) 140:883–99. doi: 10.1016/j.cell.2010.01.025 PMC2866629 20303878

[57] Goerdt S Politz O Schledzewski K Birk R Gratchev A Guillot P. Alternative Versus Classical Activation of Macrophages. Pathobiology (1999) 67:222–6. doi: 10.1159/000028096 10725788

[58] Mantovani A Sozzani S Allavena P Locati M Sica A. Macrophage Polarization: Tumor-Associated Macrophages as a Paradigm for Polarized M2 Mononuclear Phagocytes. Trends Immunol (2002) 23:549–55. doi: 10.1016/S1471-4906(02)02302-5 12401408

[59] Gordon S. Alternative Activation of Macrophages. Nat Rev Immunol (2003) 3:23–35. doi: 10.1038/nri978 12511873

[60] Liu X Zhang D Wang H Ren Q Li B Wang L. MiR-451a Enhances the Phagocytosis and Affects Both M1 and M2 Polarization in Macrophages. Cell Immunol (2021) 365:104377. doi: 10.1016/j.cellimm.2021.104377 34004369

